# Making recovery conceivable: perspectives from healthcare professionals using psychologically and behaviourally informed approaches to chronic fatigue syndrome

**DOI:** 10.1080/17482631.2026.2745089

**Published:** 2026-10-09

**Authors:** Anne Karen Bakken, Anne Marit Mengshoel, Oddgeir Synnes

**Affiliations:** a Centre for Diaconia and Professional Practice, VID Specialized University, Oslo, Norway; b Department of Public Health and Interdisciplinary Health Sciences at the Institute of Health and Society, University of Oslo, Oslo, Norway

**Keywords:** Chronic fatigue syndrome, CFS, ME, CFS/ME, recovery, treatment, healthcare professionals’ perspective, biopsychosocial model, therapeutic alliance, epistemic trust

## Abstract

**Purpose:**

Healthcare professionals (HCPs) treating patients with chronic fatigue syndrome (CFS) work within a contested field marked by uncertainty. This study explored how HCPs using psychologically and behaviourally informed approaches to CFS understood therapeutic change and their role in facilitating recovery.

**Methods:**

We used reflexive thematic analysis of two focus groups and three individual interviews with 16 HCPs from five professions, purposively sampled across clinical settings. Participants had at least two years’ experience working with patients with CFS and represented diverse therapeutic and theoretical perspectives.

**Results:**

The analysis was organised around four interrelated themes: (1) meeting patients in a “no-man’s-land”, (2) offering a new explanatory “map”, (3) navigating by the map, and (4) maintaining trust in a contested field. HCPs described offering frameworks integrating biological, psychological, and social processes. Two overlapping orientations emphasised either stress, overload, and impaired regulation or maladaptive learning, perception, and self-sustaining symptom processes. Across orientations, validation and fostering agency were central. Embodied experiences of improvement were considered important for strengthening trust and confidence that change was possible. Competing illness narratives challenged trust.

**Conclusions:**

Participating HCPs described explanatory frameworks, their application in practice, therapeutic relationships, and patients’ embodied experiences of improvement as interacting to make recovery conceivable.

## Introduction

Chronic fatigue syndrome (CFS), also referred to as myalgic encephalomyelitis (ME) or CFS/ME, is a diagnostic category defined by the presence and duration of a specified pattern of symptoms together with functional impairment (Graves et al., 2024; Nacul et al., 2021). For consistency, we use the term CFS throughout to refer collectively to these diagnostic designations. The diagnosis is not based on an established pathophysiological mechanism or biomarker, and several sets of diagnostic criteria are currently in use. Commonly used criteria include prolonged, disabling fatigue accompanied by post-exertional symptom exacerbation, sleep disturbances, cognitive impairment, and pain (Graves et al., 2024; Nacul et al., 2021). Although prevalence estimates vary depending on the criteria applied, CFS is estimated to affect around 0.5–1% of the population and is recognised as an important cause of prolonged disability in both adults and children (Lim et al., 2020).

Uncertainty and disagreement remain regarding how the illness should be understood, as well as what constitutes recovery. For healthcare professionals working with CFS, this is reflected in the absence of unified clinical guidelines and established practices for facilitating recovery, as well as a lack of consensus on whether recovery is attainable. These disagreements extend beyond questions of treatment options, their efficacy, and methodological concerns (Barry et al., 2024; Flottorp et al., 2022) to encompass more fundamental conceptual issues concerning the nature of CFS and what constitutes recovery (Adamowicz et al., 2014; Moore et al., 2021; Strand et al., 2019). They also involve differing views on explanatory models, communication practices, and ethical considerations in clinical interaction (Geraghty & Blease, 2019; Leary, 2020; Sharpe & Greco, 2019). Public discourse surrounding CFS has likewise been highly polarised, particularly around competing biomedical and biopsychosocial understandings, treatment approaches, and the relative credibility afforded to research evidence, clinical expertise, and lived experience (Blease & Geraghty, 2018; de Boer & Slatman, 2023; Karfakis, 2018; Lian & Grue, 2017).

One of the central questions in these debates concerns whether symptoms can be influenced through psychological and behavioural change, and whether therapeutic approaches supporting such processes can facilitate recovery. Current guidelines generally state that no curative or disease-modifying treatment for CFS has been established (CDC, 2024; NICE, 2021). Management is therefore primarily directed towards symptom relief, activity adjustment, energy management, and support aimed at reducing symptom exacerbation and improving quality of life (Bateman et al., 2021; CDC, 2024; Kaya et al., 2026; NICE, 2021). The British National Institute for Health and Care Excellence highlights that cognitive behavioural therapy (CBT) should not be regarded as a curative treatment and “should only be offered to support people who live with ME/CFS to manage their symptoms, improve their functioning and reduce the distress” (NICE, 2021). The current Norwegian clinical guideline offers somewhat greater support for cognitive therapy in selected patients but acknowledges the lack of evidence for curative treatment (Helsedirektoratet, 2014). Consequently, interventions that explicitly aim to facilitate recovery through psychological or behavioural change remain controversial. Criticism has centred on limited evidence of efficacy, concerns about symptom worsening, and ethical questions regarding expectations of recovery. In particular, some have argued that encouraging expectations of recovery may foster false hope or increase self-blame if recovery does not occur (Bateman et al., 2021; Geraghty & Blease, 2019; O'Leary, 2019; Thoma et al., 2023; Wormgoor & Rodenburg, 2021).

Nevertheless, some studies suggest that interventions targeting cognitive, emotional and behavioural processes may not only be well tolerated and help patients cope, but also reduce symptoms, improve functioning, and support recovery from CFS and other persistent somatic symptoms (Castell et al., 2011; Ingman et al., 2022; Khanpour Ardestani et al., 2021; Knoop et al., 2007; Löwe et al., 2024; Sharpe et al., 2022). Qualitative studies have likewise described former patients as attributing recovery from CFS to changes in behaviour, illness understanding, and ways of relating to symptoms; recovery was reported both with and without therapeutic support (Bakken et al., 2023; Ingman et al., 2025; Krabbe et al., 2023; Linnros et al., 2026). These therapeutic approaches draw on explanatory models that conceptualise symptoms as arising through dynamic interactions among biological, psychological, and social processes, thereby providing a rationale for interventions aimed at facilitating change (Henningsen et al., 2018a, 2018b; Löwe et al., 2024; Roenneberg et al., 2019; Van den Bergh et al., 2017).

Despite ongoing debates about therapeutic approaches, relatively little is known about how healthcare professionals working within these approaches understand recovery and their role in facilitating it. Previous qualitative studies have described clinicians' efforts to develop workable approaches to care by fostering collaborative, person-centred relationships and helping patients make sense of their symptoms despite ongoing uncertainty surrounding CFS (Bayliss et al., 2014; Horton et al., 2010; Wortman et al., 2022). However, these studies provide limited insight into how clinicians conceptualise therapeutic change and recovery. Clinicians’ assumptions are likely to shape the explanations, goals, and strategies they offer patients and how they support recovery, with implications for patients’ understanding of their symptoms and engagement with treatment. Their clinical experience may also provide insight into how these recovery processes are facilitated in practice. This study therefore explored how HCPs using psychologically and behaviourally informed approaches in the treatment of chronic fatigue syndrome understand therapeutic change and their role in facilitating recovery.

## Materials and methods

### Study design

This study employed a qualitative design generating data through focus groups and individual interviews with healthcare professionals. Focus groups were chosen to facilitate rich and nuanced discussions and the iterative exploration of shared understandings and differing perspectives through interaction among participants from different healthcare professions (Kitzinger, 1996; Malterud, 2012). Individual interviews were conducted to explore therapeutic experiences in greater depth and to include participants who did not wish to take part in a focus group. Combining interview methods was expected to provide in-depth individual perspectives alongside discussion of diverse views in focus groups, thereby enhancing data richness (Lambert & Loiselle, 2008). Reflexive thematic analysis, as outlined by Braun et al. (2021), was used to explore and interpret complex experiences and perspectives.

### Sampling, participants and recruitment

Potential participants were identified through professional networks, conferences, and direct contact with specialist services, and were invited to participate in the study. Participants were purposively sampled to ensure diversity in professional backgrounds, clinical settings, therapeutic approaches, and theoretical perspectives. Eligible participants had at least two years of clinical experience working with patients with CFS and used psychologically and behaviourally informed approaches in treatment.

A total of 16 HCPs (12 women, 4 men) were selected for participation. Sample size was considered in relation to information power, given the focused study aim and the purposively selected sample with experience directly relevant to the research question (Malterud et al., 2016). Thirteen HCPs took part in two focus groups, while three were interviewed individually.

The HCPs had professional backgrounds in medicine (*n* = 6), psychology (*n* = 6), physiotherapy (*n* = 1), clinical nutrition (*n* = 1) and nursing (*n* = 2). The six physicians held specialties in psychiatry (*n* = 2), paediatrics (*n* = 2), general practice (*n* = 2), internal medicine (*n* = 1), and public health medicine (*n* = 1), with some holding more than one specialty. All participants drew on experiences from clinical practice within the Norwegian healthcare system in their accounts. Their professional experience spanned somatic care, mental health services, interdisciplinary specialist teams, centres for patient education and empowerment, and private clinical practice. Most primarily treated adults, whereas four mainly worked with children and adolescents. Six participants had research experience in the field.

Within their shared broad clinical orientation, participants represented a range of therapeutic methods and theoretical perspectives. Their approaches targeted cognitive, emotional, behavioural, and physiological processes, and the interactions between them, with the aim of supporting recovery.

### Interview procedures

Focus group 1 was conducted as a physical meeting in a neutral location. Focus group 2 was held online to facilitate and ensure participation from various parts of the country. Based on the participants preferences, all individual interviews were conducted in the participants´ workplaces. Each session, including both focus groups and individual interviews, lasted approximately 90 minutes and was moderated by the first author using a shared semi-structured interview guide. The guide explored participants' understandings of recovery from CFS, how they understood and explained symptoms and recovery processes, therapeutic principles they considered important, perceived facilitators and barriers to recovery, communication with patients, and their role in supporting therapeutic change. It included broad, open-ended questions such as: “What do you consider important for recovery from CFS?”, “How do you explain symptoms and recovery to patients?”, “What do you see as your role in facilitating recovery?”, and “What challenges do you encounter in this work?” Discussions focused on general clinical principles and experiences rather than on specific therapeutic methods or techniques, and participants were encouraged to illustrate their perspectives with examples from clinical practice. By emphasising confidentiality, allowing time for introductions, and encouraging diverse perspectives, the moderator aimed to create an atmosphere of openness and comfort (Malterud, 2012; Tausch & Menold, 2016). In the focus groups, experiences and perspectives largely emerged through direct interaction and discussion among participants. Discussions were audio-recorded with participants’ consent and transcribed verbatim.

### Data analysis

Data were analysed using reflexive thematic analysis (Braun et al., 2021). Focus group and individual interview data were analysed as a single dataset rather than as separate data sources. Transcripts were read repeatedly for familiarity by the research team. In our engagement with the data, we were guided by the following questions: what healthcare professionals consider important for recovery in CFS, and how they understand and experience their role in facilitating recovery. Codes were generated through close engagement with the data and developed into broader themes reflecting patterns of shared meaning across the dataset. Themes were developed through an iterative and reflexive process of discussion and refinement within the research team throughout the analysis and writing process.

The multidisciplinary research team comprised researchers with different professional backgrounds, theoretical perspectives, and clinical experience from both somatic and mental healthcare services. All three researchers had previously collaborated on a qualitative study of recovery from CFS (Bakken et al., 2023). This prior work provided familiarity with recovery narratives while also making it important to remain reflexive about how our existing understandings might shape the interpretation of the present data. The first author had clinical experience working with patients with CFS and other persistent symptoms. The second author had clinical and research experience in the related field of fibromyalgia, together with theoretical expertise in medical and social science perspectives on recovery. The third author had methodological expertise in qualitative research and theoretical perspectives from the medical humanities, while bringing a perspective independent of prior clinical involvement in the CFS field. At the outset of the study, the researchers considered psychological and behavioural change as potentially relevant to symptom modification and recovery, while remaining open as to the extent and circumstances in which such change may be possible.

We recognised that these different backgrounds informed the questions we asked, the patterns we attended to, and our interpretations. Consistent with reflexive thematic analysis, we viewed these perspectives as interpretive resources. We deliberately questioned interpretations that aligned readily with our prior understandings and actively explored alternative readings of the data before agreeing on the final themes.

### Ethical considerations

The study was approved by the Norwegian Regional Committee for Medical and Health Research Ethics (REK) and Norwegian Agency for Shared Services in Education and Research (NSD) (REK: 192119, NSD: 342533). In accordance with Norwegian health research legislation, medical and health research projects required prior approval from the REK and NSD. All participants provided written informed consent. Confidentiality was maintained throughout the research process.

## Results

Participants’ accounts described a therapeutic process unfolding through four interrelated and partly overlapping themes. Together, these reflected a dynamic therapeutic process, moving from (1) meeting patients in a “no-man’s-land”, through (2) offering a new “map” for understanding and (3) navigating by the map: supporting change in practice, to (4) maintaining trust in a contested field: a delicate balancing act.

### Meeting patients in a “no-man’s land”

HCPs described patients as often bringing considerable emotional and experiential “baggage” from years of illness and repeated healthcare encounters. One clinician’s reflection illustrates what was frequently described as the starting point for treatment:

*“They experience themselves as physically ill and go through a long and exhausting biomedical investigation—hearts, kidneys, livers—without finding an explanation. Then they’re referred to psychiatry, where they often don’t fit traditional diagnostic categories either. They end up in this kind of in-between space, in no-man’s-land … and no one truly sees or validates what they’re going through.”*

HCPs noted that many patients described confusing and exhausting journeys through a siloed healthcare system in search of a cause for their illness. They recounted how, in previous healthcare encounters, patients were often told that they were either healthy or that the problem was “all in their minds” when no identifiable somatic disease could be found. Patients were described as feeling distrusted, dismissed, or, in some cases, stigmatised and suspected of malingering. A thorough assessment, including reassurance that no serious underlying disease had been overlooked, was considered by HCPs to be a necessary starting point for treatment, helping to reduce concerns about serious illness among both patients and clinicians. Nevertheless, HCPs described this process as often leaving an explanatory gap regarding why patients were ill and how their symptoms should be understood. While a diagnosis of CFS could provide a label and, at times, a sense of recognition, it did not offer an explanation or a clear way forward. Patients were therefore often described as feeling uncertain, confused, or anxious when informed that the cause of CFS was unknown and that no curative treatment was available. Reflecting on the limitations of previously encountered approaches, one HCP commented:

*“We can’t keep clinging to a purely biomedical model. Many still do—as if what can’t be measured simply doesn’t exist.”*

Like several others, this HCP described how reliance on a purely biomedical “map” often left patients with CFS in uncharted territory. Participants emphasised the importance of meeting patients with understanding and sensitivity to how they understood and experienced their illness. To avoid reinforcing feelings of being mistrusted or dismissed, they sought to establish a safe therapeutic relationship through humility, attentive listening, and validation of patients' experiences:

*“It’s about validating the patient’s experience: if you’re tired, you’re tired. I can’t measure that, but it’s real for you. My role is to explore plausible explanations and help make sense of the symptoms, even if their causes are not what the patient believes.”*

Uncertainty and helplessness were explicitly acknowledged, and care was taken not to question the reality of patients’ symptoms. Clinicians carefully distinguished between patients’ experiences and the explanations proposed for them. Conflating the two was described as risking mistrust, whereas maintaining this distinction allowed clinicians to validate patients’ symptoms while still exploring alternative explanations. Against this background, participants emphasised establishing sufficient trust before introducing different ways of understanding the illness.

### A new “map” for understanding

The perceived limitations of the biomedical model led HCPs to introduce a new “map” that could better bridge mind and body, help patients make sense of and navigate their illness, and make potential ways forward more visible. One HCP described how she typically spoke with patients and their families:

*“Multiple stressors can lead to exhaustion, but the body can also learn that state and recognise it. We see this with pain and fatigue: if you break your arm, for example, acute pain can develop into chronic pain that the body ‘remembers.’ A similar process can occur with fatigue following an infection.”*

The new “map” encompassed biological, psychological, and social dimensions, with HCPs placing less emphasis on CFS as a diagnosis and more on general mechanisms through which persistent symptoms could arise, be maintained, and potentially change. Across accounts, two overlapping orientations emerged: one understanding fatigue and related symptoms as signs of cumulative stress and overload, and another emphasising maladaptive learning and the brain’s role in generating and interpreting bodily signals.

From a stress- and overload-oriented perspective, symptoms could be triggered by infection or other biological events, while their development and persistence were understood as arising from an accumulation of physical, emotional, and social demands that exceeded the individual's capacity for regulation and recovery:

*“For this girl, it started with a viral infection that quickly knocked her off her feet. But as I’ve come to know the family, I can see stressors that were present long before this. She’s been running at a high gear for a long time, and within the family there’s a festering wound of shared trauma.”*

While acknowledging possible biological triggers, this kind of framing shifted attention towards the broader circumstances in which symptoms developed and persisted. By linking ongoing symptoms to patients’ own experiences and circumstances, HCPs sought to identify less visible sources of stress—including fear of serious or undetected disease—that might contribute to their persistence. Some grounded these explanations more explicitly in biological processes and terminology:

*“Many patients need recognition that what they’re experiencing is physical. Knowledge about the body’s stress systems and autonomic nervous system can offer useful and reassuring explanations, although I present it more as a mechanism than a cause.”*

In this and similar accounts, HCPs explained how stress-related processes could affect autonomic regulation and other bodily systems, including sensory, hormonal, and immune functioning. Such explanations were described as providing a coherent rationale for diverse symptoms while enhancing credibility and accessibility. Some HCPs deliberately used physiological language—and at times avoided more explicitly psychological terms—to foster reassurance, trust, and engagement with the proposed framework.

Related accounts placed greater emphasis on individual vulnerability and regulatory capacity:

*“Some of my patients are very active and driven and not attuned to their internal signals or limits… When overwhelmed, their window of tolerance narrows, everyday stress becomes unmanageable, and cognitive processes disconnect from the emotional system.”*

Here, some HCPs drew more explicitly on psychological concepts, describing difficulties recognising and regulating stress and emotions and encouraging reflection on the balance between external demands and internal needs. For some patients, bodily discomfort was also understood as potentially reflecting psychological stressors or conflicts of which they were not fully aware.

Taken together, stress- and overload-oriented explanations were used when they resonated with patients’ experiences and circumstances, making symptoms more understandable while introducing the possibility of change. The second orientation placed greater emphasis on self-perpetuating processes and the brain’s role in symptom perception and generation. Persistent symptoms were described as reflecting learning processes that increased the sensitivity of the body’s “alarm system”:

*“With long-lasting pain and fatigue, it’s like a pathway forms in the brain. Over time, that pathway becomes more well-worn, so the system becomes more sensitive—it takes less and less to trigger symptoms. You can think of fatigue as the body’s alarm system… and if that alarm becomes too sensitive, it can get stuck.”*

HCPs also described how previous experiences could shape subsequent symptoms through expectations and predictions:

“*Our expectations shape experience as much as what actually happens—that's how the brain works. From this perspective, chronic fatigue can be understood as a chronic misprediction. The brain has learned that activity is exhausting, so that prediction is triggered before you even begin, shaping the experience itself.”*

Across accounts, HCPs viewed symptoms as potentially becoming self-perpetuating through learning, sensitisation, and expectation, and increasingly decoupled from the original trigger. They considered such processes relevant even when stress had contributed to symptom development. This perspective presented persistent symptoms as understandable and potentially modifiable through new experiences and relearning, and was considered particularly useful for patients who did not identify with stress-centred explanations.

Beyond providing an explanation, a new “map” could itself have therapeutic effects. Sometimes, receiving it prompted a rapid response:

*“I always set aside a good amount of time for explanation in the first session, and patients often return saying, ‘Wow, things have really improved. Could it really be that I’m not seriously ill after all?”*

Offering an alternative framework was understood to reduce uncertainty and fear, foster hope, and make recovery appear more conceivable and attainable. However, HCPs emphasised that shifts in understanding were rarely sufficient on their own.

### Navigating by the map: supporting change in practice

Moving from insight to action, HCPs described how the new “map” was translated into practical changes tailored to the individual patient:

*“With children and adolescents, I often see them regain normal functioning once they, and often more importantly the parents - understand what this is about—and when parents and family also help reduce some of the strain.”*

Where external stressors or fear of severe disease were considered important, applying the map meant identifying and reducing key pressures. Typical examples included family or workplace conflicts and challenges at school, such as learning difficulties or bullying.

More often, however, progress depended on patients learning to navigate by the new “map”, translating it into concrete, individualised actions and testing these through experience. Many HCPs drew on principles of graded exposure and gradual re-engagement with daily activities, while emphasising that this should be guided by reducing overall strain rather than simply increasing activity:

*“I don’t find the staircase metaphor helpful. It suggests that recovery requires constant effort and struggle. For me, recovery isn’t about climbing over a hump but going under it—reducing stress rather than pushing activity. It’s about letting go of constant control; recovery becomes more of an inner journey than a climb.”*

When stress and overload were considered central, therapeutic work focused on helping patients reduce overall strain and develop a more adaptive balance between demands and restorative or pleasurable activities. Strategies for emotional and physiological regulation aimed to expand patients’ capacity to manage external pressures without becoming overwhelmed. Symptoms could therefore be treated as meaningful signals requiring attention, though not necessarily as signs of ongoing disease or a need for rest.

Within the second orientation, applying the map involved responding differently to symptoms rather than treating them as inherently meaningful signals. Therapeutic efforts focused on modifying illness beliefs, symptom-related expectations, and habitual responses through experiences intended to alter how symptoms were produced and perceived. Describing the kind of change he considered necessary for recovery, one participant explained:

*“Many patients with CFS have experienced overexertion and intense symptoms that become strongly encoded, so activity comes to predict extreme exhaustion. Gradual exposure and new experiences can challenge these expectations. It can be understood as relearning how to interpret bodily signals or resetting an overly sensitive alarm system.”*

Patients were described as commonly interpreting symptoms as signals of danger, leading to avoidance intended to prevent exacerbation. Although protective in the short term, HCPs viewed such strategies as potentially contributing to reduced functioning and poorer quality of life over time. Therapeutic work therefore involved creating new experiences in which previously avoided activities or stimuli could be encountered without being treated as dangerous. Interventions sought to “retrain the brain” by gradually reintroducing activities and stimuli associated with symptom worsening, while moderating symptom responses and reconsidering beliefs and expectations thought to contribute to symptom persistence. Through such experiences, patients could test and refine the new “map”, potentially updating expectations and reducing symptoms. Some participants referred to this as “reprogramming,” a computer analogy that patients were described as finding easy to understand and apply.

Although analytically distinguishable, the two orientations often overlapped in practice, with therapeutic strategies frequently combined or applied sequentially.

### Maintaining trust in a contested field: a delicate balancing act

HCPs described how an empathic, non-judgemental stance was often sufficient to establish an initial therapeutic relationship. Across approaches, however, they emphasised that trust ultimately depended on patients experiencing change in their own bodies:

*“…the proof of the pudding is in the eating.”*

Embodied experiences of improvement strengthened patients’ confidence in the therapeutic process and their growing sense that they could influence their own symptoms and functioning. While education and practice-based learning were considered important, experiencing change first-hand was pivotal in transforming theoretical understanding into lived experience. A central challenge in alliance-building was sustaining a collaborative and resilient relationship over time—one capable of supporting patients as they engaged with new ways of understanding their symptoms, experimented with unfamiliar practices, and tolerated uncertainty while awaiting experiential confirmation of change.

Support from family members or other caregivers was described as crucial in this process, at times reinforcing and sustaining the approach introduced in clinical encounters. One participant illustrated this through the role of a severely ill patient’s husband:

*“He had to support her because she had no balance and feared falling down the stairs. But he said, ‘No, it’s not dangerous. We’ll walk slowly—this is our training now.’ There have to be steady, reassuring people around them who believe that even if they faint, vomit, or have diarrhoea, it’s still not dangerous: ‘We set the next goal and carry on.’”*

Such support was described as essential for sustaining engagement in a demanding and often counterintuitive process of change. Close others could assist patients, moment by moment, in practising new ways of relating to their symptoms.

HCPs also noted that recovery narratives from individuals with whom patients could identify could foster hope and openness to change, sometimes marking a pivotal shift in trust in the way forward. HCPs with personal experience of recovery from CFS were considered particularly credible in this regard:

*“Some therapists have recovered from CFS themselves and bring that experience into the therapy room. It gives them unique credibility—becoming a kind of ‘living proof’ that recovery is possible.”*

At the same time, the clinician–patient relationship was described as fragile and vulnerable to multiple sources of strain. Ongoing controversies surrounding CFS and anticipated or experienced criticism from patients, families, patient organisations, colleagues, and health authorities could challenge clinicians’ confidence and sense of legitimacy:

*“If I’m met with messages like ‘Recovery isn’t possible’ or ‘The ME association says this, so you don’t know what you’re talking about,’ we’re dealing with conflicting views. That can make me feel insecure as a clinician and hesitant about being questioned or criticised. When trust is missing, it becomes much harder to offer patients the safety and hope they need.”*

HCPs described how such tensions could undermine their ability to communicate confidence and provide clear guidance. Some also expressed residual diagnostic uncertainty and concern about how current approaches might be viewed if future research were to establish a treatable biological dysfunction with little or no role for the processes they currently targeted. Several clinicians also reported being criticised for not believing patients or for minimising their suffering, highlighting the moral and professional stakes involved.

Sustaining a collaborative and trusting relationship was therefore experienced as a delicate balancing act: being sufficiently challenging to support change while avoiding loss of trust or credibility. Many addressed conflicting information and external influences explicitly in treatment, for example by discussing competing messages, recommending recovery narratives, or guiding patients towards alternative sources of support. Some also advised caution regarding certain patient organisations.

Despite these challenges, participants noted that clinical encounters were often less conflict-ridden than public discourse might suggest:

*“From my experience, it’s much easier to talk with patients than to engage in public debates… There’s a striking gap between public discourse and the reality of meeting patients in the clinic.”*

In this context, the clinician–patient relationship was described as a necessary anchor, enabling patients to remain oriented and engaged while navigating an uncertain and often contested therapeutic landscape.

## Discussion

This study explored how HCPs using psychologically and behaviourally informed approaches in the treatment of CFS understood therapeutic change and their role in facilitating recovery. HCPs described supporting recovery by offering alternative explanatory frameworks, particularly involving stress regulation and relearning, and working with patients to translate these into practice through changes in behaviour and responses to symptoms. Within this process, patients’ embodied experiences of improvement were considered important in making the new framework credible and strengthening their confidence that change was possible, thereby making recovery conceivable. A trusting therapeutic relationship was central, with validation of patients’ symptoms and suffering fostering openness to reconsidering previous interpretations of their symptoms. At the same time, HCPs described trust as vulnerable within a clinical context marked by uncertainty, contested knowledge, and competing explanatory models (Figure 1). These findings suggest that a meaningful understanding of symptoms, its translation into practice, patients’ experiences of change, and the therapeutic relationship may all be important considerations when developing and delivering psychologically and behaviourally informed treatment for CFS.

**Figure 1.**
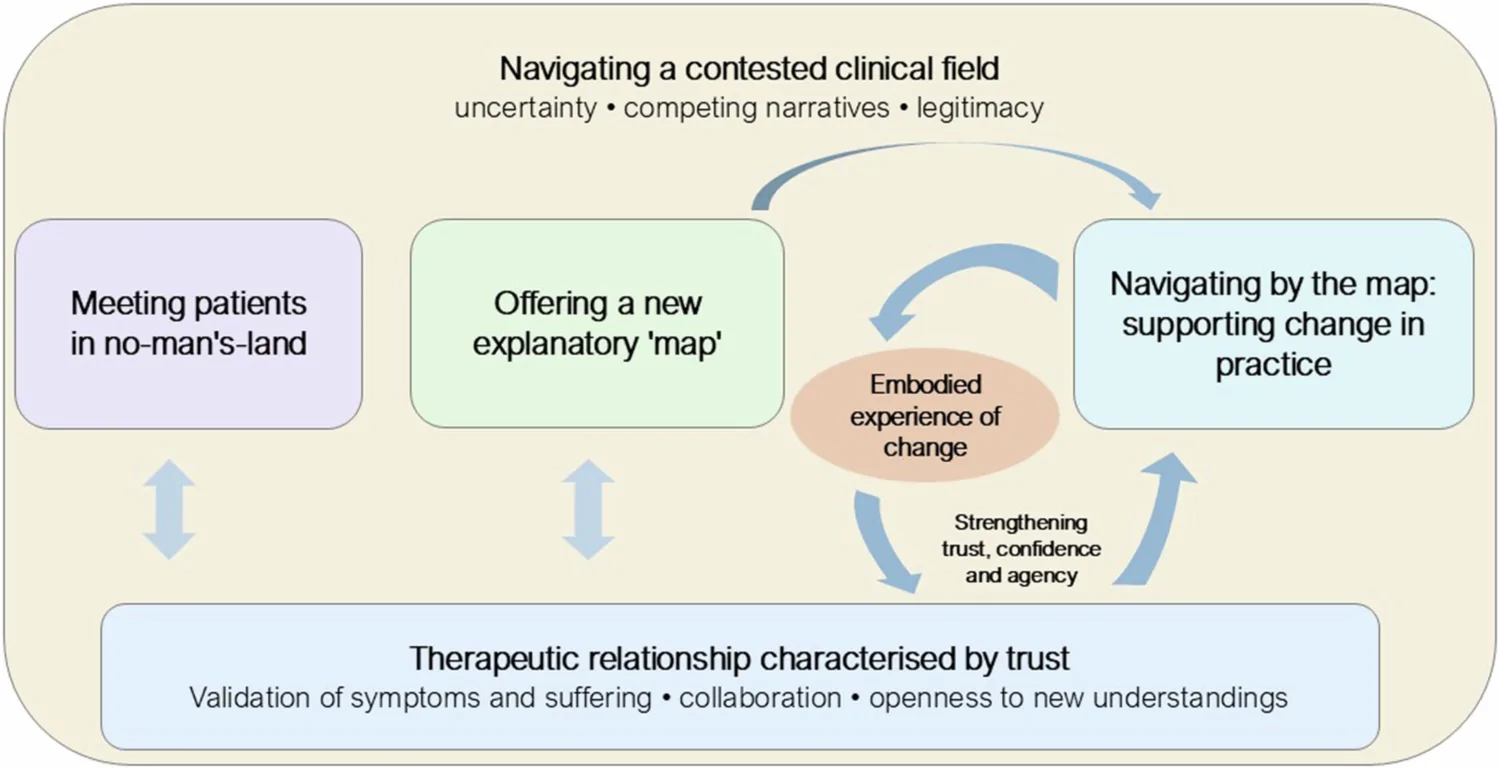
Conceptual relationships among themes describing how participating healthcare professionals supported recovery from CFS. Figure note: Conceptual relationships among the four themes. The model illustrates the authors' interpretation of how participating healthcare professionals described supporting recovery from CFS. Arrows indicate the authors' proposed conceptual relationships and influences among themes rather than causal pathways.

In the following discussion, we interpret these findings through three interrelated lenses: explanatory frameworks as therapeutic “maps”, the therapeutic relationship and epistemic trust as conditions for therapeutic change, and the challenges of navigating a contested clinical and societal context.

### The role of explanatory frameworks as therapeutic “maps”

A central feature of these explanatory “maps” was that their value appeared to lie less in resolving questions of causation than in providing orientation for change by identifying potentially modifiable processes. The explanatory frameworks described by this purposively selected group of HCPs broadly reflected a biopsychosocial orientation, emphasising interactions among biological, psychological, behavioural, and social processes in symptom persistence and recovery. Importantly, this did not represent a simple shift from “biological” to “psychological” explanations, but from what HCPs described as a more biologically determined view of symptoms towards one in which symptoms and the processes contributing to their persistence were understood as dynamic and potentially modifiable. In this respect, these frameworks differed from a traditional biomedical disease model, in which treatment is primarily aimed at identifying and targeting underlying pathology (Hewa & Hetherington, 1995; Hofmann, 2005). Participating HCPs viewed a predominantly biomedical understanding of CFS as potentially fostering a more fixed view of symptoms and offering fewer avenues for therapeutic change. This interpretation resonates with previous qualitative studies of former patients’ recovery narratives, in which shifts towards more dynamic and modifiable understandings of symptoms were described as fostering agency and providing a sense of direction (Bakken et al., 2023; Hannah Linnros et al., 2026). The convergence between HCP and former-patient perspectives suggests that shifts in illness understanding may be relevant to therapeutic change and recovery, providing a basis for hypotheses that could be explored in future research.

Within this broadly shared biopsychosocial framework, we identified two overlapping explanatory orientations of symptom persistence and recovery. One foregrounded stress and overload, with therapeutic efforts directed towards reducing strain and strengthening regulatory capacity; the other emphasised learning and brain processes involved in symptom perception. Both can be situated within contemporary integrative models of persistent physical symptoms, which incorporate interacting biological, psychological, behavioural, and social processes, including stress-related processes, learning, and symptom perception (Henningsen et al., 2018b; Kube et al., 2020; Löwe et al., 2024). Predictive processing provides an account of how prior experience and expectations may shape symptom perception through processes operating largely outside conscious awareness. This is particularly relevant to the orientation emphasising brain processes and learning. Here, relearning was understood as involving changes not only in how patients interpret or respond to bodily signals, but also in how symptoms themselves are generated and experienced. The two ways of understanding identified in our study should be regarded as HCPs’ clinical interpretations rather than as established mechanisms underlying CFS or recovery. Despite their different emphases, both orientations shared a therapeutic focus extending beyond symptom management to symptom reduction, improved functioning, and the possibility of recovery.

The findings also highlight potential challenges in applying these frameworks. Explanations centred on altered or misleading bodily signals may help make sense of symptoms in the absence of identifiable ongoing pathology, but could also lead patients to discount bodily sensations that warrant medical attention or further investigation. At the same time, although reducing stress and overload may be helpful, this may not in itself be sufficient to resolve persistent symptoms. Approaches focused primarily on changing interpretations, expectations, or learned responses may likewise be insufficient where ongoing stressors and regulatory challenges contribute to symptom persistence.

Previous critiques of biopsychosocial approaches in CFS have highlighted the importance of incorporating patients’ narratives and avoiding explanations experienced as invalidating or overly psychological (Geraghty & Blease, 2019). Rather than representing competing or comprehensive explanatory models, the two orientations may offer complementary perspectives whose relevance depends on the individual patient and clinical context. Other theoretical frameworks may offer different interpretations of symptom persistence and recovery; evaluating competing explanatory models was beyond the scope of the present study.

### Therapeutic relationship and epistemic trust

The importance that participating HCPs attached to the therapeutic relationship resonates with psychotherapy research on the therapeutic alliance, commonly conceptualised as agreement on treatment goals and tasks together with a personal bond (Bordin, 1979). Therapeutic alliance has consistently been associated with better outcomes in psychotherapy across a range of mental health conditions (Baier et al., 2020; Flückiger et al., 2018; Martin et al., 2000), with similar associations reported in other healthcare contexts (Kinney et al., 2020). Wampold and Budge (2012) further proposed that, once an initial bond is established, therapeutic change involves the real relationship, the development of expectations, and engagement in health-promoting actions. Although relatively understudied in CFS and other persistent somatic symptoms, existing research suggests that the therapeutic relationship may be relevant to therapeutic change and recovery in these conditions (Heins et al., 2013; Wortman et al., 2022). In CFS specifically, positive outcome expectations and early alliance formation have been associated with improvement during cognitive behavioural therapy (Heins et al., 2013).

Our findings, however, suggest that the role of trust extended beyond establishing a collaborative therapeutic alliance. Participating HCPs repeatedly emphasised that understanding new explanations was insufficient on its own; patients also needed embodied experiences that lent credibility to the proposed framework in practice. We interpret this pattern through the concept of epistemic trust, referring to the extent to which therapeutic input is experienced as credible, personally relevant, and applicable. Epistemic trust has been proposed as a central component of effective therapy, enabling patients to take up and apply new knowledge (Fonagy & Allison, 2014). Its development is thought to depend on patient-related factors, the therapeutic relationship, and the therapist’s ability to tailor communication and interventions to the patient’s needs and level of epistemic trust (Fisher et al., 2025). To our knowledge, epistemic trust has not been specifically examined in relation to recovery from CFS. Viewed through this lens, HCPs’ accounts portrayed embodied experiences of improvement as strengthening patients’ trust in the proposed framework and in their own capacity to influence symptoms, thereby making recovery conceivable.

### Navigating a contested field

Participating HCPs described supporting recovery within a contested clinical and societal context that could challenge both the therapeutic relationship and the epistemic trust involved in therapeutic change. Competing narratives framing CFS as a severe biomedical condition, alongside critiques of psychological or psychiatric framings—including concerns about stigmatisation, invalidation, and harmful treatment—have been widely documented (Dumit, 2006; Geraghty & Blease, 2019; Shapiro & Hayburn, 2024; Spandler & Allen, 2018). The concept of epistemic injustice (Fricker, 2007) has been applied to healthcare encounters involving patients with CFS (Blease et al., 2017; Spandler & Allen, 2018). Testimonial injustice may occur when patients’ accounts are afforded insufficient credibility, while hermeneutical injustice arises when adequate shared concepts or frameworks for making sense of illness experiences are lacking (Kidd & Carel, 2017). Patients with CFS have been described as vulnerable to both (Byrne, 2020), and biopsychosocial approaches have themselves been criticised as potentially contributing to epistemic injustice when they override patients’ illness understandings or minimise bodily suffering (Blease et al., 2017). Conversely, de Boer (2021) has argued that patient participation dominated by biomedical understandings may itself constrain which forms of knowledge and understandings of illness are considered legitimate.

Our findings add complexity to this debate. HCPs portrayed shifts in patients’ illness understanding as potentially important, but also challenging, with much at stake for both patients and clinicians. Participants emphasised patients’ fear of symptom exacerbation and the uncertainty involved in interpreting symptoms in new ways. For clinicians, perceived risks extended beyond the therapeutic relationship to professional legitimacy, criticism from colleagues and the public, regulatory scrutiny, and possible legal repercussions.

Against this background, HCPs described carefully negotiating how alternative explanations were introduced and applied in order to preserve trust while supporting change. In doing so, they navigated a delicate balance between validation and reappraisal, hope and realism, and agency and blame. By offering a “map” oriented towards the present and potential ways forward, rather than towards identifying a single root cause, HCPs sought to provide direction for the therapeutic process. Viewed through the lens of epistemic injustice, these accounts highlight a tension: reframing symptoms may risk invalidating patients’ existing understandings, yet alternative frameworks may also help patients make sense of their symptoms in ways that support agency and make recovery conceivable.

## Strengths and limitations

A strength of this study lies in the inclusion of HCPs from diverse professional backgrounds, clinical settings, and therapeutic approaches, providing a broad range of experience relevant to the study aim. Combining focus groups and individual interviews provided complementary perspectives, richness, and nuance. The quality of the analysis was supported by a transparent and reflexive analytic process involving ongoing discussion and refinement of interpretations, alongside critical consideration of how the research team’s assumptions and perspectives shaped the analysis.

Participants were purposively recruited because they worked with therapeutic approaches based on the premise that psychological and behavioural change could contribute to symptom improvement and recovery in CFS. Although the sample was considered sufficient in terms of information power, its relatively small size may have limited the breadth of perspectives represented. This sampling strategy was chosen to address the study aim and should be considered when interpreting the findings. Consequently, the findings reflect the perspectives of HCPs working within this particular clinical orientation rather than the full range of views among healthcare professionals working with CFS. Likewise, the patients described by participating HCPs may have been particularly engaged, motivated, or receptive to these therapeutic approaches, potentially contributing to an underrepresentation of more critical perspectives and limiting the transferability of the findings to other patient populations. The study does not address the effectiveness of these approaches or the experiences of patients who do not improve or who experience deterioration, which warrant investigation from patients’ perspectives.

Furthermore, the Norwegian specialist healthcare context may limit transferability to other healthcare systems and cultural settings. Given the contested nature of the field, some participants may also have moderated or withheld views during the focus groups despite efforts to encourage open discussion of differing perspectives. Although the findings cannot be assumed to represent all healthcare professionals working with CFS, the diversity of professional backgrounds and clinical settings represented suggests that the analysis captures issues likely to be relevant in similar clinical contexts.

## Conclusion

This study contributes to understanding how therapeutic change and recovery may be conceptualised and supported within a clinical field characterised by uncertainty and competing explanatory models. Drawing on the clinical experiences of a purposively selected group of HCPs, the findings point to an interplay between explanatory frameworks, their application in practice, trusting therapeutic relationships, and patients’ embodied experiences of improvement, rather than locating therapeutic change in any single explanation or technique. Viewed through the lens of epistemic trust, these accounts suggest how trusting relationships and experienced improvement may lend credibility to new ways of understanding and responding to symptoms, thereby helping make recovery conceivable. At the same time, the findings highlight the relational and ethical challenges of sustaining trust within a contested clinical field. Further research should explore patients’ experiences of these approaches and their different elements, including how they relate to therapeutic relationships, trust, and recovery over time.

## Data Availability

Due to privacy and ethical restrictions, the qualitative data (interview transcripts) supporting the findings of this study are not publicly available.
